# Assessing quality of life in childhood cancer survivors at risk for hearing loss: a comparison of HEAR-QL and PROMIS measures

**DOI:** 10.3389/fonc.2024.1362315

**Published:** 2024-03-06

**Authors:** Anne Spence, Allison J. L’Hotta, Susan S. Hayashi, Kara Felts, Emily LaFentres, Megan Jones-White, Judith E. C. Lieu, Allison A. King, Robert J. Hayashi

**Affiliations:** ^1^ Department of Pediatrics, Division of Pediatric Hematology Oncology, Washington University in St. Louis, St. Louis, MO, United States; ^2^ Brown School, Prevention Research Center, Washington University in St. Louis, St. Louis, MO, United States; ^3^ Department of Otolaryngology, Division of Pediatric Otolaryngology, Washington University in St. Louis, St. Louis, MO, United States

**Keywords:** QOL, childhood cancer survivor, ototoxicity, hearing loss, HEAR-QL, PROMIS

## Abstract

**Background:**

Childhood cancer survivors (CCS) exposed to platinum chemotherapy are at an increased risk of developing hearing loss and reporting decreased quality of life (QOL). This study compared two QOL measures; one developed for children with hearing loss, The Hearing Environments and Refection on Quality of Life (HEAR-QL), and one validated in CCS, the Patient-Reported Outcomes Measurement Information System (PROMIS), to assess their ability to evaluate QOL deficits in this population.

**Methods:**

Subject eligibility were restricted to CCS exposed to platinum-based chemotherapy but who were free of known risk factors for cognitive impairment, (non-central nervous system tumor, no cranial radiation, or intrathecal chemotherapy). Participants had to be between 8-17 years, have completed anti-cancer therapy for at least 6 months, and have an audiogram within 1 year, Participants completed the HEAR-QL-26 (7-12 years) or the HEAR-QL-28 (13-18 years) and the PROMIS. Independent samples and/or one sample T-tests were utilized to compare participants with normal hearing and hearing loss, and to compare outcome measures to normative HEAR-QL and PROMIS data. Non-parametric correlations were utilized to evaluate the relationship between QOL and demographic and medical variables, and QOL and severity of hearing loss.

**Results:**

Fifty-four CCS were evaluable. The mean age was 12.0 years. Twenty-eight participants (51.9%) received cisplatin, 30 (55.6%) carboplatin, and 4 (7.4%) received both. Twenty participants (37%) demonstrated hearing loss. Participants with hearing loss scored significantly lower on the HEAR-QL than those with normal hearing (mean: 70.3, SD: 21.7, vs mean: 88.0, SD: 9.3, p =.004 for the HEAR-QL-26; mean: 84.7, SD: 10.2 vs mean: 94.8, SD: 3.4, p =.040 for the HEAR-QL-28). Participants with normal hearing scored significantly lower on the HEAR-QL-26 than the normative mean (mean: 88, SD: 9.3, normative mean: 98, SD: 5, p =.000). The PROMIS failed to identify any differences in QOL between participants based on hearing status, or when compared to the normative mean.

**Conclusion:**

The HEAR-QL was more sensitive than the PROMIS in identifying QOL deficits in CCS at risk for hearing loss. The HEAR-QL should be considered in studies seeking to improve the QOL of CCS with hearing loss.

## 1 Introduction

Understanding the late effects of cancer therapy has become increasingly important as the 5-year survival rate for the pediatric cancer population currently exceeds 80% (1, 2). Platinum-based chemotherapy agents such as cisplatin and carboplatin are routinely used in treatment, and although they continue to be highly effective chemotherapy agents, they are also associated with the development of significant late effects, among which include ototoxic hearing loss and decreased quality of life (QOL) (3–6). There is evidence to suggest that the development of ototoxic hearing loss and decreased QOL share similar risk factors; such as, younger age at diagnosis, central nervous system (CNS) malignancies, and exposure to cranial radiation, making it difficult to assess whether they are independent outcomes (7, 8). The majority of research regarding QOL in childhood cancer survivors (CCS) has not utilized measures that specifically examine the impact of hearing loss, and thus it remains unclear how hearing relates to QOL in CCS. Thus, we proceeded to evaluate QOL in patients at risk for hearing loss utilizing two measures: the PROMIS, which has been validated in pediatric cancer patients, and the HEAR-QL, which was specifically developed for children with hearing loss, to compare how each measure assessed QOL in CCS (9, 10).

## 2 Materials and methods

This was a pilot study of pediatric oncology patients followed in the Division of Hematology and Oncology at St. Louis Children’s Hospital and St. Louis Children’s Specialty Care Center. The Human Research Protection Office’s (HRPO) Institutional Review Board (IRB) at the Washington University School of Medicine, and the Protocol Review and Monitoring Committee at the Siteman Cancer Center in St Louis approved study protocol. This study was supported by a grant through the St. Louis Children’s Hospital, Washington University School of Medicine, Children’s Discovery Institute (CDI, MC-II-2019-779 King, PI).

The population of this study was designed to exclude known conditions and treatments which have been shown to be associated with CNS toxicity as many of these variables have been shown to be associated with impaired QOL (11–14). Exclusion criteria included a history of a CNS malignancy, prior radiation therapy to the head, exposure to intrathecal chemotherapy, a history of a baseline neurocognitive or psychological disorder, or if the parent and/or patient were unable to read English. Eligible subjects and their parent/guardians were approached by a study team delegate to participate during a routine Late Effects Clinic visit. Eligible participants had a prior diagnosis of a non-CNS pediatric solid tumor, had been exposed to ototoxic chemotherapy agents (cisplatin and/or carboplatin), were between the ages of 8-17 years due to age restrictions of the assessment tools, had completed anti-cancer therapy for at least 6 months, had completed an audiogram with good to fair reliability within 1 year of enrollment and were English speaking. Participants and parents/guardians signed informed consent and/or assent approved by the Human Research Protection Office’s (HRPO) Institutional Review Board (IRB). Compensation was provided in the form of a $50 gift card for participation in the study, but all participants enrolled on a voluntary basis. Medical record review was completed on enrolled participants to obtain the following variables of interest; gender, age, race and ethnicity, diagnosis, age at diagnosis, exposure to cisplatin and/or carboplatin with cumulative dose, exposure to aminoglycosides (gentamicin, tobramycin, and/or amikacin) with or without toxic level documented, radiation exposure to any part of the body and dosage, end of treatment date, hearing status, toxicity grades according to the International Society of Pediatric Oncology (SIOP), and whether or not a participant had been previously fit with hearing aids.

### 2.1 Audiologic methods

All participants completed routine audiologic evaluations as standard care. These evaluations were conducted by licensed audiologists at St. Louis Children’s Hospital or the St. Louis Children’s Specialty Care Center. Behavioral hearing tests were conducted in a sound-treated booth utilizing a clinical audiometer with TDH30 headphones or ER-3A inserts as transducers. Since all participants were between 8-17 years of age, they were tested according to conventional audiometric testing techniques. Participants were required to have an audiogram with good to fair reliability within one year of enrollment.

Audiometric thresholds were evaluated from 250-8,000 Hz. Normal hearing was defined as thresholds ≤ 20 dB HL across frequencies. Sensorineural hearing loss (SNHL) was defined as air conduction pure tone thresholds at any frequency ≥ 25 dB HL accompanied by bone conduction thresholds within 10 dB of the air conduction thresholds. Tympanometric results were considered normal when compliance was ≥ 0.2 mmhos, or when there was a large ear canal volume consistent with patent pressure equalization tubes.

In this study, audiograms were classified by the SIOP grading scale, which is emerging to be the preferred scale for evaluating populations of pediatric cancer patients and CCS. The SIOP scale is intended for monitoring hearing during treatment, and has been found to be an easy to use, clinically applicable scale for classifying ototoxic hearing loss, and therefore may be used in making recommendations for patient management (15, 16). SIOP classifications refer to a patient’s documented bone line, which is typically evaluated from 500-4000 Hz (17). When there was a hearing loss identified above 4,000 Hz (6,000 or 8,000 Hz) that could not be defined by bone conduction, air conduction thresholds were used to determine the SIOP grade only if tympanometry results were within normal limits. SIOP grades were documented for both ears in order to fully capture the degree and impact of ototoxic hearing loss. **Supplementary Table 1** displays the parameters distinguishing between SIOP grades. SIOP grades range from 0 to 4, with grade 0 signifying normal hearing and lower to higher SIOP grades (1-4) signifying a gradually increasing severity of hearing loss. Participants were considered to have a hearing loss when they had a SIOP score in either ear that was greater than a grade 0, in order to capture the impact of both unilateral and bilateral hearing losses.

### 2.2 Quality of life measures

Participants were asked to complete the Hearing Environments and Reflection on Quality of Life (HEAR-QL). The HEAR-QL is a quality of life questionnaire developed in the Department of Otolaryngology at Washington University School of Medicine in St. Louis. The HEAR-QL is a self-reported quality of life questionnaire specifically designed to assess how a child or adolescent perceives the impact of his or her hearing loss across various QOL domains. Subjects who utilize a hearing aid are instructed to complete the questionnaire based on how they hear while utilizing amplification. Two versions of the HEAR-QL were utilized in this study; the HEAR-QL-26 which is intended for children ages 7-12 years, and the HEAR-QL-28 which is intended for adolescents ages 13-18. The HEAR-QL-26 consists of 26 questions addressing three QOL domains; Environments, Activities, and Feelings. The HEAR-QL-28 consists of 28 statements addressing four QOL domains; Hearing Situations, Social Interactions, School Difficulties, and Feelings. Both the HEAR-QL-26 and HEAR-QL-28, utilizes a five-point scale with answer choices including; Never (4), Almost Never (3), Sometimes (2), Often (1), and Almost Always (0). These scores are transformed to a 0-100-point scale; Never (100), Almost Never (75), Sometimes (50), Often (25) and Almost Always (0). Scores closer to 100 indicate a higher self-perceived quality of life related to hearing. Mean scores are provided for each individual domain and for the total scale score. A total scale score of 98 and 95 for the HEAR-QL-26 and HEAR-QL-28 respectively, have been established as the normative mean values based on validation studies of children with both normal hearing and hearing loss (10, 18).

Participants additionally completed the PROMIS, the Patient-Reported Outcomes Measurement Information System (PROMIS), a quality of life questionnaire initiated by the National Institutes of Health (NIH). See *www.NIHPROMIS.org* for additional information (19). The PROMIS was created to provide researchers and clinicians access to standardized patient-reported QOL measures, that have been validated in multiple diverse patient groups, including in children with cancer (20–22). The PROMIS Pediatric measures are intended for use as self-report measures, in children and adolescents between 8 and 17 years of age. Scores are reported as standardized T-scores, with a mean of 50 and a standard deviation of 10. A higher T-score indicates that more of a certain concept is being measured, which can be desirable or undesirable depending on the measure of interest.

This study assessed quality of life utilizing the Pediatric Profile – 49, which is comprised of seven unique pediatric QOL measures; PROMIS Ped SF v2.0 – Mobility 8a, PROMIS Ped SF v2.0 – Anxiety 8a, PROMIS Ped SF v2.0 – Depressive Symptoms 8a, PROMIS Ped SF v2.0 – Fatigue 8a, PROMIS Ped SF v2.0 – Peer Relationships 8a, PROMIS Ped SF v2.0 – Pain Interference 8a, and the PROMIS Pain Intensity Item (9033R1r), and utilized two additional short forms, the PROMIS Ped SF v1.0 – Cognitive Function 7a and the PROMIS Ped Scale v2.0 – Anger 9a (23–26). Therefore, in this study, higher T scores for measures assessing mobility, cognitive function, and peer relationships were considered desirable, while higher T-scores for measures assessing anxiety, anger, pain, depressive symptoms, and fatigue were considered undesirable.

### 2.3 Data collection

Study data were collected and managed using REDCap electronic data capture tools hosted at Washington University School of Medicine (27, 28). REDCap (Research Electronic Data Capture) is a secure, web-based software platform designed to support data capture for research studies, providing 1) an intuitive interface for validated data capture; 2) audit trails for tracking data manipulation and export procedures; 3) automated export procedures for seamless data downloads to common statistical packages; and 4) procedures for data integration and interoperability with external sources. The HEAR-QL-26 and the HEAR-QL-28 were adapted into a REDCap survey for participant completion with permission from Washington University School of Medicine. Auto-scoring PROMIS measures (Pediatric Profile – 49, PROMIS Ped SF v1.0 – Cognitive Function 7a, and the PROMIS Ped Scale v2.0 – Anger 9a) were downloaded from the shared REDCap library (29). Participants completed all QOL measures at their own pace on a clinic iPad, with their parent/guardian and a study representative present.

### 2.4 Statistical methods

Descriptive statistics were compiled for participant demographics and outcome measures. Independent samples t-tests were completed to compare outcome measures for participants with normal hearing to participants with hearing loss with a significance level of 0.05. One-sample t-tests were completed to compare outcomes measures to established normative means. Non-parametric correlations (Spearman’s rho) were completed to evaluate the relationship between QOL (HEAR-QL Total Scale Scores and PROMIS domains) and various demographic and medical continuous variables (age at diagnosis and testing, time since diagnosis, time since treatment completion, and total dose of cisplatin [mg/m^2^], carboplatin [mg/m^2^] and radiation [cGy]. Differences in QOL based on cancer type and severity of hearing loss, determined by SIOP grade (0-4), were assessed using the non-parametric Kruskal-Wallis test. Nonparametric statistics were used because the underlying distribution of the sample did not follow a normal distribution.

## 3 Results

Fifty-seven participants were enrolled in this study. Three participants were excluded from the analysis; one withdrew from the study and two did not complete study measures. Ultimately, 54 participants were evaluable in the study. See **Table 1** for participant demographics, treatment, and hearing characteristics. Evaluable participants had a mean age of 3.8 years (range 0-15 years) at the time of their cancer diagnosis and had a mean age of 12.0 years at time of enrollment. Participants were on average, 8.08 years (range 1-16.7 years) from their diagnosis and on average 7.13 (range 0.7 -16.2 years) from end of treatment. There were 29 (53.7%) males and 25 (46.3%) females in the study population. Neuroblastoma (29.6%), germ cell tumor (24.1%), and retinoblastoma (18.5%) made up the majority of diagnoses in this cohort. Twenty-eight participants (51.9%) received cisplatin, 30 (55.6%) carboplatin, and 4 (7.4%) received both agents. Five participants (9.4%) were exposed to aminoglycosides with 1 (20%) of those participants having toxic level documented. Twelve (22.6%) participants received radiation to some location in the body other than the head.

**Table 1:** Participant demographic, treatment, and hearing characteristics (n= 54).

Characteristic	n (%) or mean (range; SD)				
Gender					
Male	29 (53.7)				
Female	25 (46.3)				
Age and Time					
Age in years (mean)	12.0 (8-17; SD 2.99)				
Age in years at diagnosis	3.80 (0-15; SD 4.22)				
Time in years since diagnosis	8.08 (1-16.7, SD 11.10)				
Time in years since end of treatment	7.13 (0.7 -16.2, SD 10.96)				
Race					
Caucasian	50 (92.6)				
African American	2 (3.7)				
Asian	1 (1.9)				
Other	1 (1.9)				
Diagnosis					
Neuroblastoma	16 (29.6)				
Germ cell tumor	13 (24.1)				
Retinoblastoma	10 (18.5)				
Osteosarcoma	5 (9.3)				
Wilms tumor	4 (7.4)				
Hepatoblastoma	4 (7.4)				
Clear cell carcinoma of kidney	2 (3.7)				
Treatment					
Cumulative Cisplatin Dose (mg/m^2^) (n = 27) *	469.70 (134.15-800; SD 164.42)				
Cumulative Carboplatin Dose (mg/m^2^) (n= 31)	2633.51 (560-9350; SD 1697.04)				
Aminoglycoside Exposure (n=53)** Toxic Level Documented (n=5)	5 (9.4) 1 (20)				
Received Radiation (n=53) **	12 (22.6)				
Location of Radiation (multiple sites allowed)					
Abdomen	11 (20.4)				
Chest	4 (7.4)				
Groin	1 (1.9)				
Pelvis	2 (3.7)				
Not applicable	41 (75.9)				
Mean Total Dosage of Radiation (n = 12)	2899.71 (1080-6116.5; SD 1416.44)				
Hearing					
SIOP Grade	0	1	2	3	4
Lower SIOP grade	36 (66.7)	6 (11.1)	6 (11.1)	5 (9.3)	1 (1.9)
Higher SIOP grade	34 (63.0)	3 (5.6)	6 (11.1)	10(18.5)	1 (1.9)
Symmetric SIOP grades	47 (87)				
Asymmetric SIOP grades***	7 (13)				
Bilateral hearing loss	18 (33.3)				
Unilateral hearing loss	2 (3.7)				
Hearing aid use					
Bilateral	8 (40)				
Unilateral	2 (10)				

*n =27 due to missing data for one participant treated at an outside facility.

**n= 53 due to missing data for one participant treated at an outside facility.

*** = SIOP grades are different for the two ears.

Twenty participants (37%) demonstrated sensorineural hearing loss: eighteen (33.3%) participants demonstrated bilateral hearing loss and two participants (3.7%) demonstrated unilateral hearing loss. Thirteen (72%) of the patients with bilateral hearing loss had the same SIOP grade in both ears (symmetrical hearing loss). Eight (40%) of the twenty participants with hearing loss were previously fit with bilateral hearing aids, and two (10%) were fit with a unilateral hearing aid. Of note, the two participants who utilized a unilateral hearing aid each had a bilateral hearing loss (lower SIOP grade of 1) and utilized the hearing aid in their ear with the higher SIOP grade (SIOP grade 2, SIOP grade 3). The two participants with unilateral hearing loss did not utilize a hearing aid in their ear with the higher SIOP grade (SIOP grade 1, SIOP grade 3). The 10 participants who had been previously fit with hearing aids were required to wear them for the duration of the study. Of the 28 participants who received cisplatin, 20 (71.4%) had hearing loss while, 3 of the 30 (10%) participants who received carboplatin had hearing loss, however, these three participants received both carboplatin and cisplatin.

All participants completed the HEAR-QL (see **Table 2**); 31 participants completed the HEAR-QL-26, with 13 (41.9%) experiencing ototoxic hearing loss and 23 participants completed the HEAR-QL-28, with seven (30.4%) experiencing ototoxic hearing loss. Participants with hearing loss had significantly lower total scale scores on the HEAR-QL (mean: 70.3, SD: 21.7 vs mean: 88.0, SD: 9.3, p =.004 for the HEAR-QL-26; mean: 84.7, SD: 10.2 vs mean: 94.8, SD: 3.4, p = .040 for the HEAR-QL-28) than participants with normal hearing. Significant differences were also demonstrated for CCS with hearing loss compared to those with normal hearing on the Feelings subscale (mean: 67.6, SD: 26.3 vs mean: 91.5, SD: 11.8, p=.008 for the HEAR-QL-26; mean: 79.9, SD: 16.4 vs mean: 96.9, SD: 3.2, p =.034 for the HEAR-QL-28). Participants with hearing loss had worse scores across the Environments subscale on the HEAR-QL-26 than participants with normal hearing (mean: 62.3, SD: 25.3 vs mean: 81.6, SD: 14.0, p =.023). Furthermore, participants with hearing loss had worse scores than those with normal hearing on the HEAR-QL-28 for the Hearing Situations subscale (mean: 64.9, SD: 21.6 vs mean: 85.4, SD: 10.2, p =.047).

Table 2Differences in HEAR-QL scores based on hearing status.

**Table:** 

HEAR-QL-26 (n=31)	Normal Hearing Mean (SD) n= 18	Hearing Loss Mean (SD) n= 13	p-value
Environments subscale	81.6 (14.0)	62.3 (25.3)	0.023
Activities Subscale	97.9 (5.9)	90.7 (20.9)	0.248
Feelings Subscale	91.5 (11.8)	67.6 (26.4)	0.008
Total Scale Score	88.0 (9.3)	70.3 (21.6)	0.004

**Table:** 

HEAR-QL-28 (n=23)	Normal Hearing Mean (SD) n= 16	Hearing Loss Mean (SD) n= 7	p-value
Hearing Situations Subscale	85.4 (10.2)	64.9 (21.6)	0.047
Social Interactions Subscale	99.8 (0.9)	100.00 (0.0)	0.521
School Difficulties	95.3 (6.5)	91.8 (8.2)	0.286
Feelings Subscale	96.9 (3.2)	79.9 (16.4)	0.034
Total Scale Score	94.8 (3.6)	84.7 (10.2)	0.040

**Figure 1:**
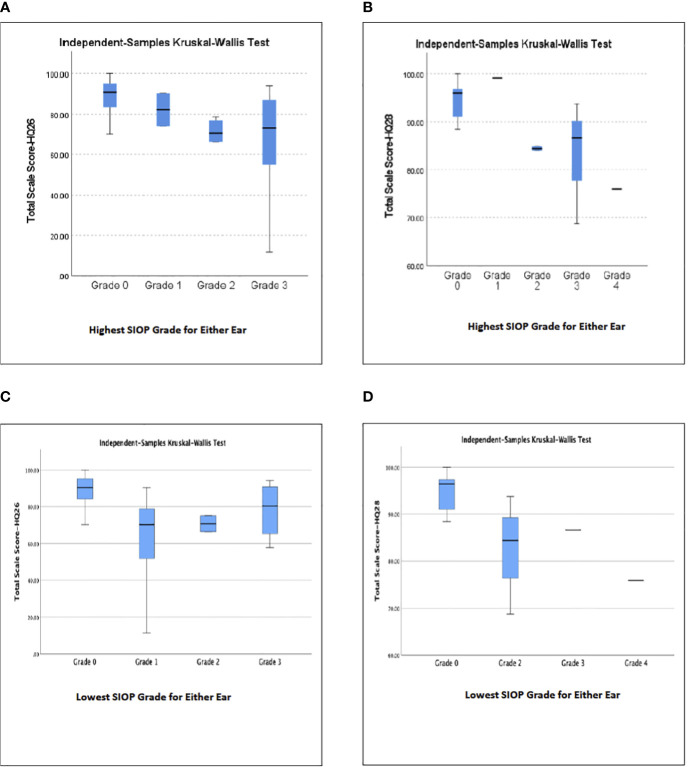
Relationship of subjects’ highest **(A, B)** or lowest **(C, D)** SIOP Grade of hearing loss in either ear to Total Scale Score QOL for **(A, C)** HEAR-QL-26, **(B, D)** HEAR-QL-28.

**Figure 2:**
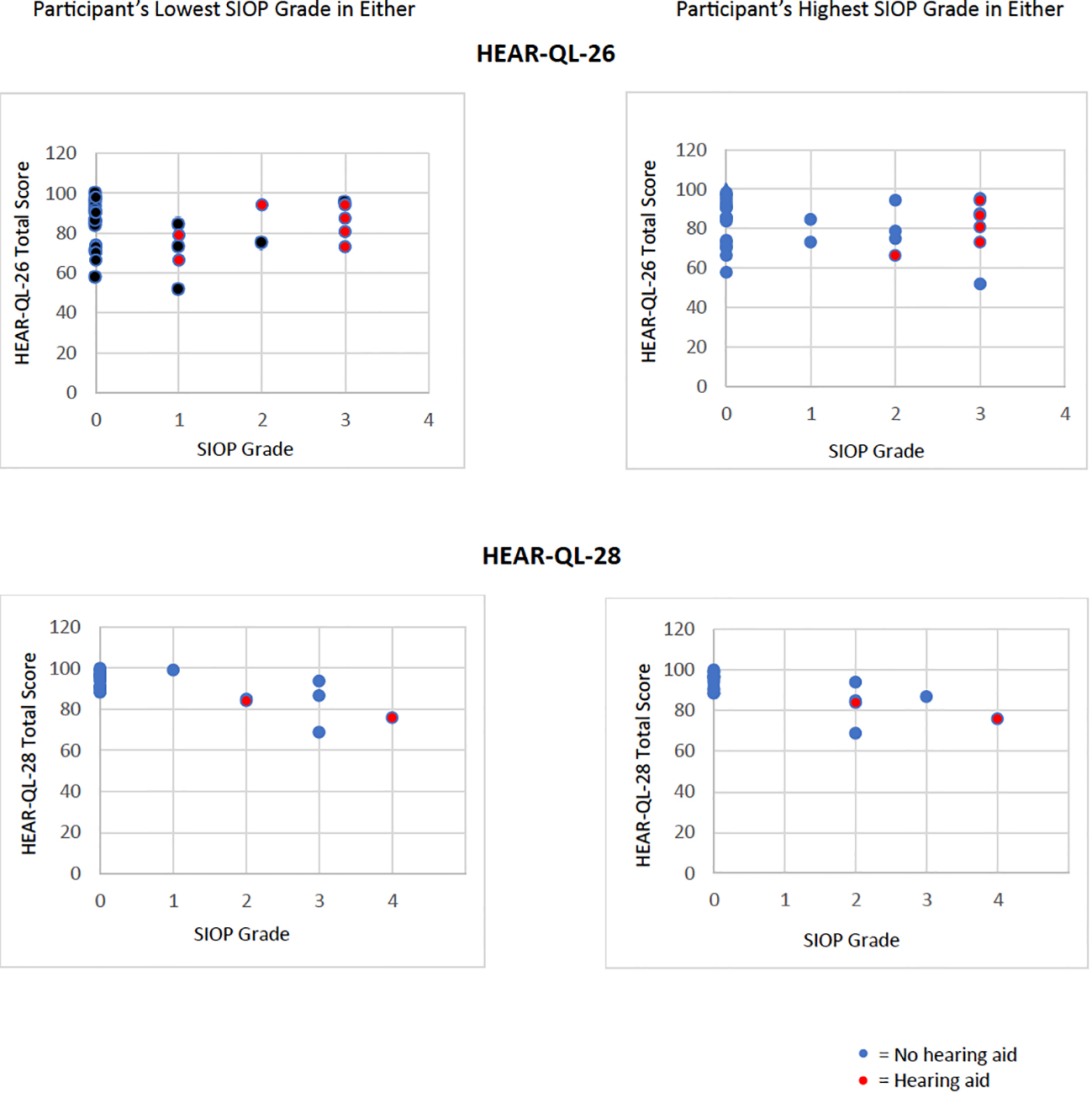
Hearing aids compensate the impact of increasing SIOP grades on worsening QOL. Data of Total Scale Score QOL from HEAR-QL-26 and HEAR-QL-28 plotting against the participant’s lowest or highest SIOP grades for either ear. Red dots represent participants who were wearing hearing aids at the time of evaluation .

A majority of participants in this study, whether they had hearing loss or normal hearing, had the same, or symmetric SIOP grades in each ear, however, seven participants (13%) had different, or asymmetric SIOP grades in each ear. Analysis of the Total Scale Scores from the HEAR-QL-26 and HEAR-QL-28 both demonstrated significant differences between the HEAR-QL Total Scale Score and SIOP grade when analyzing for the ear with the patient’s higher SIOP grade (HEAR-QL-26 p=.038, HEAR-QL-28 p=.022), or their lowest SIOP grade (HEAR-QL-16 p= 0.027), HEAR-QL-28 p= 0.016), See **Figure 1**). However, none of the Pairwise comparisons were significant, presumably due to the small sample size. There was a trend to suggest that participants who had previously been fit with hearing aids reported higher QOL than participants with hearing loss who had not been fit with hearing aids. (See **Figure 2**). This suggests that hearing aid utilization may correlate with improved QOL.

Participant’s HEAR-QL Total Scale Scores were compared to the established normative means. Participants with normal hearing (mean: 88, SD: 9.3, normative mean: 98, SD: 5, p =.000) and with hearing loss (mean: 70.3, SD: 21.6, normative mean: 98 SD: 5, p = .001) scored significantly lower than the normative mean on the HEAR-QL-26. There was not a significant difference between the HEAR-QL-28 Total Scale Scores for participants with normal hearing and the normative mean, however, participants with hearing loss did score significantly lower than the normative mean (mean: 84.7, SD: 10.2, normative mean: 95, SD: 8, p = .037).

The PROMIS did not detect significant differences between QOL in normal hearing participants or participants with hearing loss in this population. There were no significant QOL deficits identified in this population of CCS when PROMIS scores were compared to the normative mean (50). In examining other variables that may have influences hearing loss, we observed that ten (18.5%) of the participants had a diagnosis of retinoblastoma, with none of those participants demonstrating hearing loss. This is in contrast to our other majority diagnosis groups, neuroblastoma and germ cell tumor, where six out of the sixteen (37.5%) participants with neuroblastoma demonstrated hearing loss and six out of the thirteen (46.2%) participants with germ cell tumor demonstrated hearing loss. Furthermore, we reviewed each subject’s past record for other ototoxic exposures and identified 5 patients with aminoglycoside exposure with only one participant demonstrating a drug level in the toxic range and that participant had normal hearing. These and additional variables (age, gender, race, diagnosis, age at diagnosis, time in years since diagnosis, time in years since end of treatment, and treatment related variables including cumulative dosing of cisplatin or carboplatin, and total dosage of radiation) were examined in relation to participant reported QOL. None of these variables impacted the relation of hearing loss to the observed QOL outcomes.

## 4 Discussion

This study demonstrated that the HEAR-QL was more sensitive than the PROMIS in identifying QOL deficits in CCS who are at an elevated risk for ototoxic hearing loss but at low risk for other CNS associated late effects. The HEAR-QL identified impairments in QOL related to hearing status within this population of CCS and when compared to the normative mean. Even normal hearing participants ages 7-12 in this cohort scored lower than the normative mean, potentially suggesting that the HEAR-QL can even detect the impact of cancer therapy on QOL who are at risk for hearing loss but who have normal audiologic evaluations. CCS at risk for hearing loss are on strict ototoxic monitoring schedules at our institution, and therefore the subjects may be more aware and/or knowledgeable about potential challenges related to hearing loss or may be experiencing changes in their hearing that are not routinely monitored, including changes in extended high-frequency (9000-16000 Hz) hearing and/or in speech-in-noise understanding following the exposure to ototoxic agents (30, 31).

The PROMIS failed to identify any differences in QOL either with participants with normal hearing or participants with ototoxic hearing loss. The PROMIS has been previously validated in populations of CCS. However, we specifically investigated a population of non-CNS cancer survivors and therefore eliminated traditional variables that have been shown to have a negative impact on the CNS including cranial radiation and/or intrathecal chemotherapy. If ototoxic hearing loss is one of the primary late effects for non-CNS cancer survivors treated with platinum chemotherapy agents, then a measure specifically geared toward evaluating the impact of hearing loss on QOL, such as the HEAR-QL, may be a more effective measure in this population.

### 4.1 Strengths and limitations

This study utilized two different QOL surveys to assess self-reported QOL in pediatric non-CNS cancer survivors. Administering both QOL surveys has added value to improve our understanding of differences in different QOL measures in CCS as the PROMIS has previously been validated in pediatric cancer patients and the HEAR-QL has previously been validated in non-cancer populations of children with normal hearing and hearing loss. To our knowledge, this study was the first to administer the HEAR-QL to a population of CCS. In populations of pediatric cancer survivors with hearing loss, it is important to utilize QOL measures that are sensitive to factors relating to both cancer treatment and hearing impairment and which correlate to the severity of ototoxic hearing impairment and QOL. Since ototoxic chemotherapeutic agents such as cisplatin and carboplatin are used often in the treatment of pediatric cancer patients, assessing the QOL related to hearing may be of considerable value to assist this population. Of note, 18.5% of the participants in this study had a diagnosis of retinoblastoma. Although CCS with retinoblastoma are at risk for hearing loss, no participants with retinoblastoma had hearing loss in this cohort. Specific data relating to vision and/or visual deficits was not analyzed in this study, however, it is reasonable to assume that a decrease in a participant’s vision could also have an impact on quality of life, which has been reported (32–34). This project did not detect any difference in QOL based on diagnosis, however, in CCS with hearing and vision impairment, the combined sensory deficits may have an even greater impact on QOL. Fortunately, retinoblastoma patients are typically treated with carboplatin which is typically associated with less ototoxicity in contrast to patients with neuroblastoma and germ cell tumor patients who experienced ototoxicity (35). Future investigations should examine the potential of a unique and/or compounding impact of both vision and hearing deficits on the QOL of CCS.

The PROMIS is an established, valid, and reliable tool to use with CCS. However, for the purposes of this study, the framing of some of the questions and statements on the PROMIS measures may have impacted survey responses in this population. The measures asked participants to respond to each question and statement in the context of, “*In the past 7 days”* with the exception of the Cognitive Function domain that asked participants to respond to each question or statement in the context of “*In the past 4 weeks.*” A potential explanation for why participants did not have QOL scores significantly decreased when compared to the normative values, could be due to the time frame provided by the PROMIS measures. CCS who have completed anti-cancer treatment for a minimum of 6 months could be displaying resiliency and could be rating their current QOL in comparison to their QOL while actively undergoing treatment.

The scope of this study did not allow for assessments of QOL at multiple timepoints, or for the assessment of QOL at baseline. When QOL is assessed throughout the cancer experience, self-reported measures have been found to vary and fluctuate throughout treatment and recovery (36). Approaching patients at a singular point during their cancer experience may not holistically depict the QOL of a CCS and therefore it is important to interpret the results accordingly. This study cannot make an informed statement regarding QOL before, during, and/or after cancer treatment or at the onset of a patient’s treatment related hearing loss; this study simply provides data relating to how the QOL in this population of pediatric non-CNS cancer survivors compares to normative values and that the PROMIS measure failed to display clinically significant deficits in QOL compared to the general population, regardless of their hearing status. Future investigations of QOL and hearing loss should be conducted prospectively and include the outcomes of routine audiologic evaluations to assess for any possible overlap in the onset of hearing loss and decreased QOL.

Although basic information regarding participant hearing aid use was obtained, it was beyond the scope of the study to evaluate or interpret factors relating to hearing aid benefit such as hours of day per use, verification of fitting to prescriptive targets, or functional outcome measures such as aided testing. Participants in this study who utilized hearing aids were required to wear them throughout administration of test materials, however the present study does not attempt to make definitive statements regarding individual hearing aid benefit. Therefore, the direct impact of hearing aid use could not be evaluated as a factor relating to QOL in this study. Future investigations should evaluate the impact of hearing aid use and benefit on QOL for individuals with ototoxic hearing loss.

## 5 Conclusion

Pediatric non-CNS cancer survivors experience deficits in QOL even in the absence of traditional CNS toxic exposures such as radiation to the head/neck, brain tumor and/or brain surgery, and intrathecal chemotherapy. Participants with ototoxic hearing loss have worse self-reported QOL as determined by the HEAR-QL than those with normal hearing. The HEAR-QL is sensitive to severity of hearing loss and identifies unique aspects of QOL in this patient population that other QOL measures, or those at risk for hearing loss, may not be able to evaluate. QOL screenings should be implemented in this patient population in order to connect patients with necessary services and improve outcomes in pediatric non-CNS cancer survivors. Specifically, the HEAR-QL should be considered in studies seeking to understand and improve the QOL of CCS with hearing loss.

## Data availability statement

The raw data supporting the conclusions of this article will be made available by the authors, without undue reservation.

## Ethics statement

The studies involving humans were approved by The Human Research Protection Office’s (HRPO) Institutional Review Board (IRB) at the Washington University School of Medicine, and the Protocol Review and Monitoring Committee at the Siteman Cancer Center in St Louis. The studies were conducted in accordance with the local legislation and institutional requirements. Written informed consent for participation in this study was provided by the participants’ legal guardians/next of kin.

## Author contributions

AS: Conceptualization, Data curation, Investigation, Writing – original draft, Writing – review & editing. AL’H: Formal analysis, Writing – review & editing. SH: Conceptualization, Writing – original draft, Writing – review & editing. KF: Data curation, Investigation, Writing – review & editing. EL: Investigation, Writing – review & editing. MJ-W: Writing – review & editing. JL: Writing – review & editing. AK: Formal analysis, Writing – review & editing, Data curation, Funding acquisition. RH: Conceptualization, Writing – original draft, Writing – review & editing.
