# Supplementary material for: Assessing quality of life in childhood cancer survivors at risk for hearing loss: a comparison of HEAR-QL and PROMIS measures
Source: Front Oncol. 2024 Mar 6;14:1362315. doi: 10.3389/fonc.2024.1362315 (PMC10951079; doi:10.3389/fonc.2024.1362315)
Supplement: Supplementary file 1 [file Table_1.pdf]

Supplemental Table 1: SIOP Boston Ototoxicity Scale*

Grade	Parameters
0	≤ 20 dB HL at all frequencies
1	> 20 dB HL (ie, 25 dB HL or greater) SNHL above 4000 Hz (ie, 6 or 8 kHz)
2	> 20 dB HL SNHL at 4000 Hz and above
3	> 20 dB HL SNHL at 2000 or 3000 Hz and above
4	> 40 dB HL (ie, 45 dB HL or more) SNHL at 2000 Hz and above

*Brock PR, Knight KR, Freyer DR, Campbell KCM, Steyger PS, Blakley BW, et al. Platinum-induced ototoxicity in children: A consensus review on mechanisms, predisposition, and protection, including a new International Society of Pediatric Oncology Boston ototoxicity scale. Journal of Clinical Oncology. 2012 Jul 1;30(19):2408–17. doi: 10.1200/JCO.2011.39.1110
